# Near and Not-So-Dear TRI Facilities: Prenatal Proximity and Later Brain Cancer

**Published:** 2006-07

**Authors:** Valerie J. Brown

The most clearly established environmental risk factor for childhood brain cancer is therapeutic radiation exposure (not including diagnostic X-rays). New research now suggests that children of mothers who lived near an EPA Toxics Release Inventory (TRI) facility while pregnant may be more likely to later develop brain cancer, especially if the site released carcinogens **[*EHP* 114:1113–1118; Choi et al.]**.

Prenatal exposure to chemicals can have profound long-term effects, as some toxic chemicals that are stopped by the blood–brain barrier in adults may reach the fetus via the placenta. This work is the first to specifically examine brain cancer risk in children and potential exposure to TRI releases, although some previous research has suggested slight increases in risk for certain birth defects associated with such emissions.

Of the more than 650 toxic chemicals listed in the TRI, 193 are known or suspected carcinogens, according to the EPA. Fifty-five known, probable, or possible carcinogens were actually released within 2 miles of the study participants. However, it is very difficult to accurately assess exposure to TRI releases. The TRI itself shows only the type and mass of chemicals released in a given year, not where the chemicals went or precisely when they were released. Because of the uncertainty built into using these data, studies such as this must be interpreted with caution.

The study included 382 children diagnosed with brain cancer before age 10 and an equal number of cancer-free controls analyzed as pairs. Mothers of children whose brain cancer was diagnosed before age 10 years were nearly 50% more likely to have lived within 1 mile of such a site during pregnancy; the likelihood was nearly 75% higher for children diagnosed before age 5. However, when looking at risk for two major childhood brain cancer types in particular, astrocytoma and primitive neuroectodermal tumors, there was no difference.

The team used EPA Region III’s chronic toxicity index, which combines total mass released with toxicity factors including carcinogenic weight of evidence and cancer potency factors. For this study, inhalation and oral cancer potency factors were included. Other potential factors, such as mothers’ exposures in the work-place during pregnancy, children’s postnatal exposure, and exposure through contaminated drinking water, were not taken into account. The authors therefore caution that their results are not conclusive, but should be replicated and expanded using improved exposure measures.

## Figures and Tables

**Figure:**
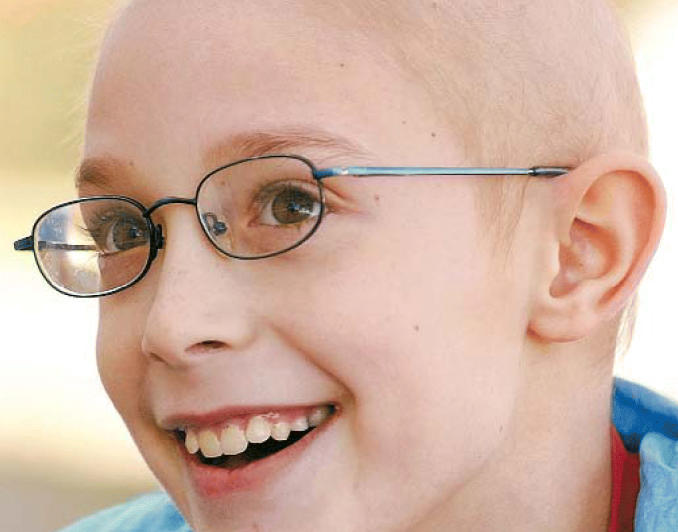
Location equation New data point to a mother’s residence near a Toxics Release Inventory facility while pregnant as a possible factor in brain cancer risk in children.

